# Point-of-care ultrasound and Doppler ultrasound evaluation of vascular injuries in penetrating and blunt trauma

**DOI:** 10.1186/s13089-017-0060-5

**Published:** 2017-02-16

**Authors:** Miguel Angel Montorfano, Fernando Pla, Leonardo Vera, Omar Cardillo, Stefano Geniere Nigra, Lisandro Miguel Montorfano

**Affiliations:** 1 Department of Ultrasound, Clemente Álvarez Emergency Hospital, Av. Pellegrini 3205, 2000 Rosario, Santa Fe Argentina; 2 Emergency Medicine Residency, University of Pavia, Strada Nuova 65, 27100 Pavia, Italy

**Keywords:** Doppler sonography, Color flow Duplex Doppler, Critical ultrasound, Point-of-care ultrasound, Vascular injury, Trauma

## Abstract

**Background:**

The aim of this study is to describe point-of-care ultrasound and Color flow Duplex Doppler characteristics of penetrating and blunt trauma-related vascular injuries of the limbs and neck.

**Methods:**

Penetrating and blunt trauma-related vascular injuries such as vein disruption, intimal flap, deep vein thrombosis, arterial dissection, pseudoaneurysm, and arteriovenous fistulae are discussed in this manuscript. Images of the most significant lesions of our personal clinical experience are presented to illustrate point-of-care ultrasound and Color flow Duplex Doppler ultrasound findings.

**Results:**

Penetrating and blunt trauma-related vascular injuries represent a big challenge. While patients with hard signs of arterial damage must be sent immediately to surgical exploration, when there are soft signs or no clear signs of vascular injury at the physical examination, and the patient is stable, imaging investigation and observation can be useful in the diagnosis and management of these patients. Although angiography is the gold standard of the imaging methods, point-of-care ultrasound and Color flow Duplex Doppler ultrasound are widely available, cheaper, noninvasive, and faster to obtain. They can provide bedside valuable information for the identification of some vascular injuries allowing to an integrated management of the trauma patient, enriched by the use of ultrasound.

**Conclusions:**

Point-of-care ultrasound and Color flow Duplex Doppler examination are increasingly used in the decision making process of trauma-related vascular injuries.

## Background

Injuries are an important public health problem worldwide. More than 5 million people die each year as a result of injuries. This accounts for 9% of the world deaths, nearly 1.7 times the number of fatalities that result from HIV/AIDS, tuberculosis, and malaria combined. Among the causes of injury are traffic accidents, interpersonal violence, burns, drowning, falls, and poisonings. A quarter of the deaths are the result of traffic injuries, while suicide and homicide account for nearly another quarter.

For every person who died as a result of violence, many more are injured, causing a rise in costs in health care systems and in a massive burden in national economies [1]. Moreover, violence exacts a high cost on global development [2].

More than 90% of injury-related deaths occur in low- and middle-income countries and in remote locations.

Increase in urban crime and violence are affecting the lives of millions of people in Latin America.

The Pan American Health Organization called violence in Latin America “the social pandemic of the 20th century” [3].

Social inequality and substantial increase in the size of drug markets are considered major contributing factors to the levels of violence in Latin America. Of the world’s 50 most dangerous cities, 43 are located in Latin America and the Caribbean.

Although the main goal must be to prevent injuries and violence from happening, a quick and rapid diagnosis and treatment are essential to prevent fatalities and reduce disabilities [1].

**Fig. 1:**
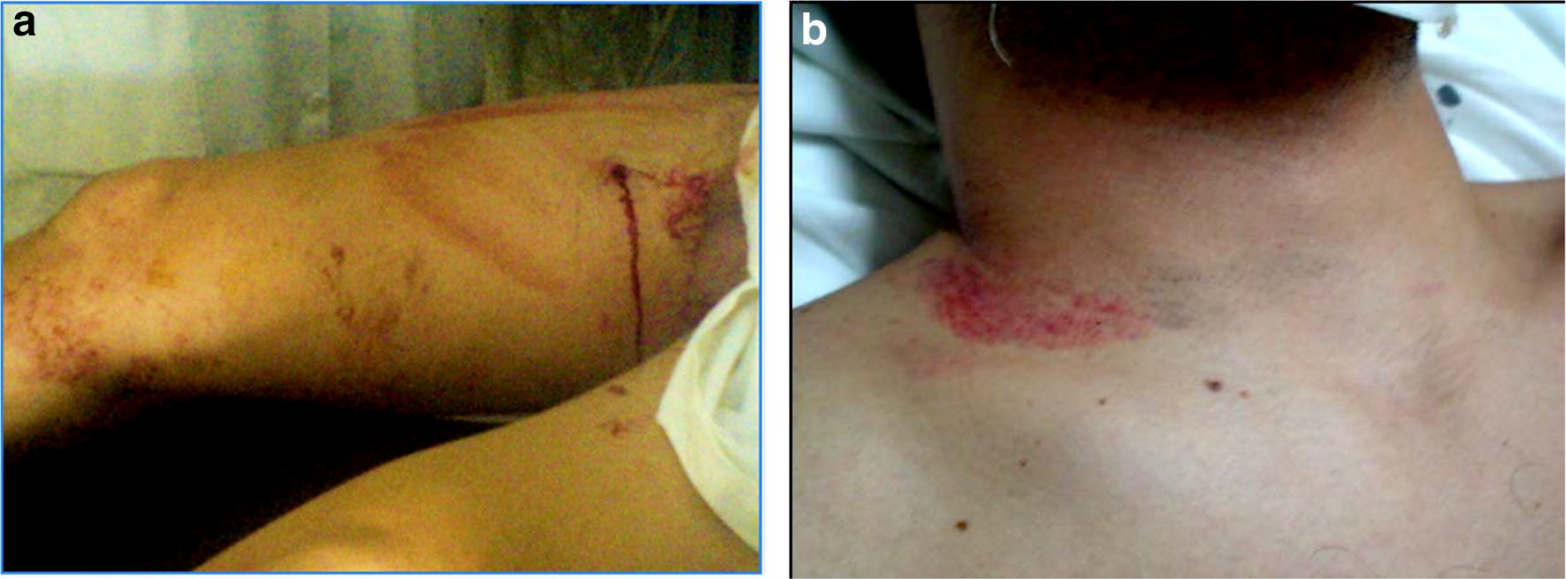
**a** Penetrating wound in the leg: gun shot wound. **b** Blunt trauma of the neck

Vascular injuries represent less than 3% of all traumatic findings, yet they are associated with potentially severe complications [4–9]. If not recognized and treated fast, injuries to major arteries and veins may have devastating consequences resulting in amputations or even death. Knowing the mechanism of the trauma is of extreme importance for making the correct diagnosis. Although blunt trauma is a common cause of acute trauma presentation in the civil population worldwide, the increasing urban violence in our region has produced a rise of penetrating trauma produced by gun shot and stab wounds that deserve especial considerations (Fig. 1). Most vascular injuries are caused by penetrating trauma, such as gunshot wounds, stabbing, and blast injuries [6]. Blunt trauma causes significant soft tissue injury, while vascular disorders are less frequent [4]. Nonetheless, also in blunt trauma, vascular injuries need to be ruled out, especially in patients with displaced long bone fractures, crush injuries, and patients with prolonged immobilization [4–7]. Blunt trauma is more often associated with partial or complete vascular occlusion, arterial dissections, and intimal flaps, while penetrating trauma can also cause pseudoaneurysms, arteriovenous fistulas, and vein thrombosis [10–13].

The majority of vascular injuries are located in the extremities. Vascular injuries present a great challenge to emergency physicians because some vascular lesions may not be immediately identified with clinical evaluation and monitoring of vital signs.

The presence of “hard signs” in the physical examination” (such as pulsatile external bleeding, expanding haematoma, absent distal pulses, cold/pale limb, palpable thrill, or audible bruit) is an indication of immediate surgical intervention.

When the site or presence of a vascular injury is less obvious, the patient is stable and there are no signs or just soft signs of vascular injuries (such as peripheral nerve deficit, history of moderate hemorrhage at scene, a reduced but palpable pulse, or an injury in proximity to a major artery) imaging investigation and observation can be useful in the diagnosis and management of these patients. [4, 14].

Angiography is the gold standard for the evaluation of trauma-related vascular injuries; however, other noninvasive imaging methods such as computer tomography angiogram, magnetic resonance angiography, and Doppler ultrasound have emerged in recent years as valid alternatives [15].

Color flow Duplex Doppler is a widely available and reliable method with high sensitivity (95–97%), specificity (95–98%), and accuracy (98%) in assessment of peripheral vascular injuries [15, 16].

Bergstein et al. compared Color flow Doppler with arteriography in 67 patients without obvious vascular injuries. Using arteriography as the gold standard, Color flow Doppler had a specificity of 99%, sensitivity of 50%, and negative and positive predictive values of 66 and 7%, respectively [16].

Fry et al. described 100% sensitivity and specificity of Duplex Doppler compared with the conventional arteriography and operative exploration [17].

In a recent study, Mohd Wani et al. evaluate with Color flow Duplex Doppler 150 patients with soft signs of vascular injury of the limbs. The patients with features of vascular injury on Color Doppler (110 patients) were subjected to exploration, while patients who had normal Doppler (40 patients) were subjected to CT- angiography. Color flow Doppler had a sensitivity of 94% and specificity of 82.5% in diagnosis of vascular injury compared with CT angiography and surgical exploration [18].

Point-of-care ultrasound is widely available in trauma centers, emergency departments and is increasingly being used in the prehospital setting and in remote locations. In many cases, it is the first diagnostic method available. In these scenarios, it is used for a whole body examination, from head to toe, allowing to an integrated management of the trauma patient [19].

In the extremities and neck Point of care ultrasound is used for the diagnosis of fractures, hematomas, and the identification of foreign bodies [20]. At the same time we propose that, in stable patients without hard signs of vascular damage, it could be of great help for a prompt bedside diagnosis, characterization, and monitoring of trauma-related vascular injuries.

### The technique

Vascular injuries of the extremities and neck must be investigated with high-frequency linear transducers because a higher resolution and a lower penetration are commonly needed. There are some exceptions, such as patients with large hematomas and super obese patients, in which a convex probe with higher penetration is recommended. When evaluating vascular injuries, we have to explore the region of the trauma, and transversal and longitudinal scans of the vessels should be performed with B-mode. Proximal and distal flows should be investigated with Color flow Duplex Doppler ultrasound. For comparative purposes, both limbs and both sides of the neck should be evaluated.

Normal veins usually are oval in shape and completely compressible with a controlled pressure of the probe. The pulsed wave Doppler analysis shows a continuous flow pattern with respiratory variations.

Normal arteries are round in shape, with an echogenic wall, and noncompressible with the probe. Arteries of the extremities show a triphasic pattern in the pulsed Doppler spectrum. The triphasic pattern has a first positive phase related with the systole of the left ventricle, a second negative phase related with the closure of the aortic valve, and a third positive phase produced by the elasticity of the arterial walls. The loss of this triphasic pattern is always pathologic [21, 22]. The absence of triphasic pattern can be acute (traumatic injury or obstruction) or chronic (atherosclerosis, diabetes, aging).

Although in trauma patients, the presence of obesity, large hematomas, subcutaneous air, or large open wounds of the skin can represent a technical limitation to the penetration of the sound waves, with point-of-care ultrasound and Color flow Duplex Doppler is possible to accurately rule in or rule out trauma-related vascular injuries, [15, 16, 23, 24].

The aim of this study is to review and describe point-of-care ultrasound and Doppler ultrasound characteristics of penetrating and blunt trauma-related vascular injuries of the limbs and neck, such as vein rupture, intimal flap, deep vein thrombosis, arterial dissection, pseudoaneurysm, and arteriovenous fistulae. A bedside identification of these lesions can help to a better care of these patients. Images of most significant lesions are presented to illustrate point-of-care ultrasound and Doppler ultrasound findings.

#### Pathologic sonographic patterns

In patients with clinical suspicion of a vascular injury, point-of-care ultrasound and Doppler ultrasound are used to answer 2 basic questions:

1. Does the patient have a vascular lesion? Yes or no?
2. If the answer is yes: what kind of lesion?

**Fig. 2:**
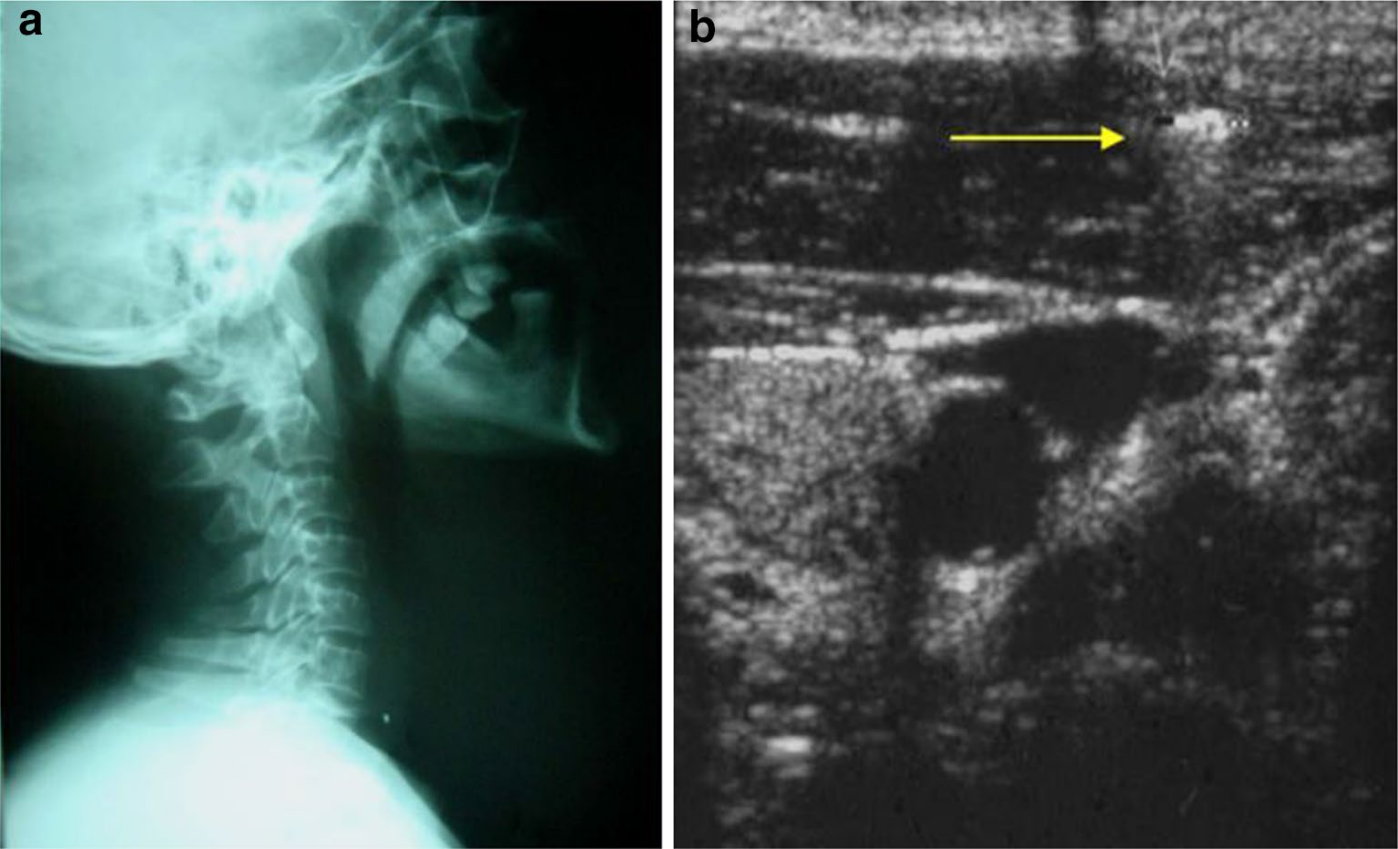
No vascular compromise: B mode. **a** X ray showing a bullit in the base of the neck. **b** Mode showing the bullit in the esternocleidomastoid muscle. No vascular compromise of the jugular vein nor the carotid artery

**Fig. 3:**
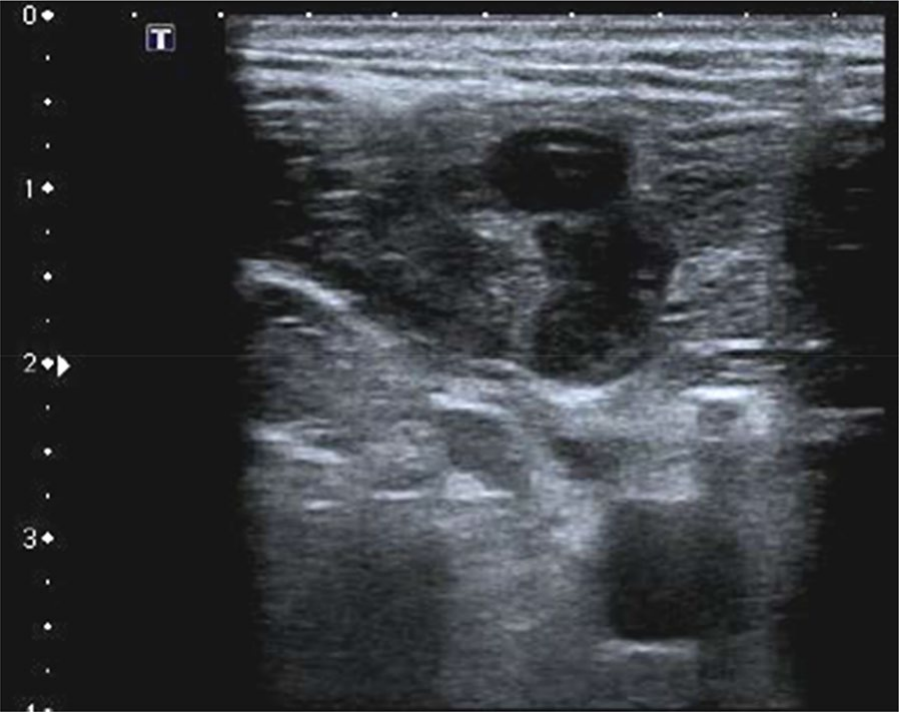
Soft tissue hematoma of the left side of the neck. No vascular injury

**Fig. 4:**
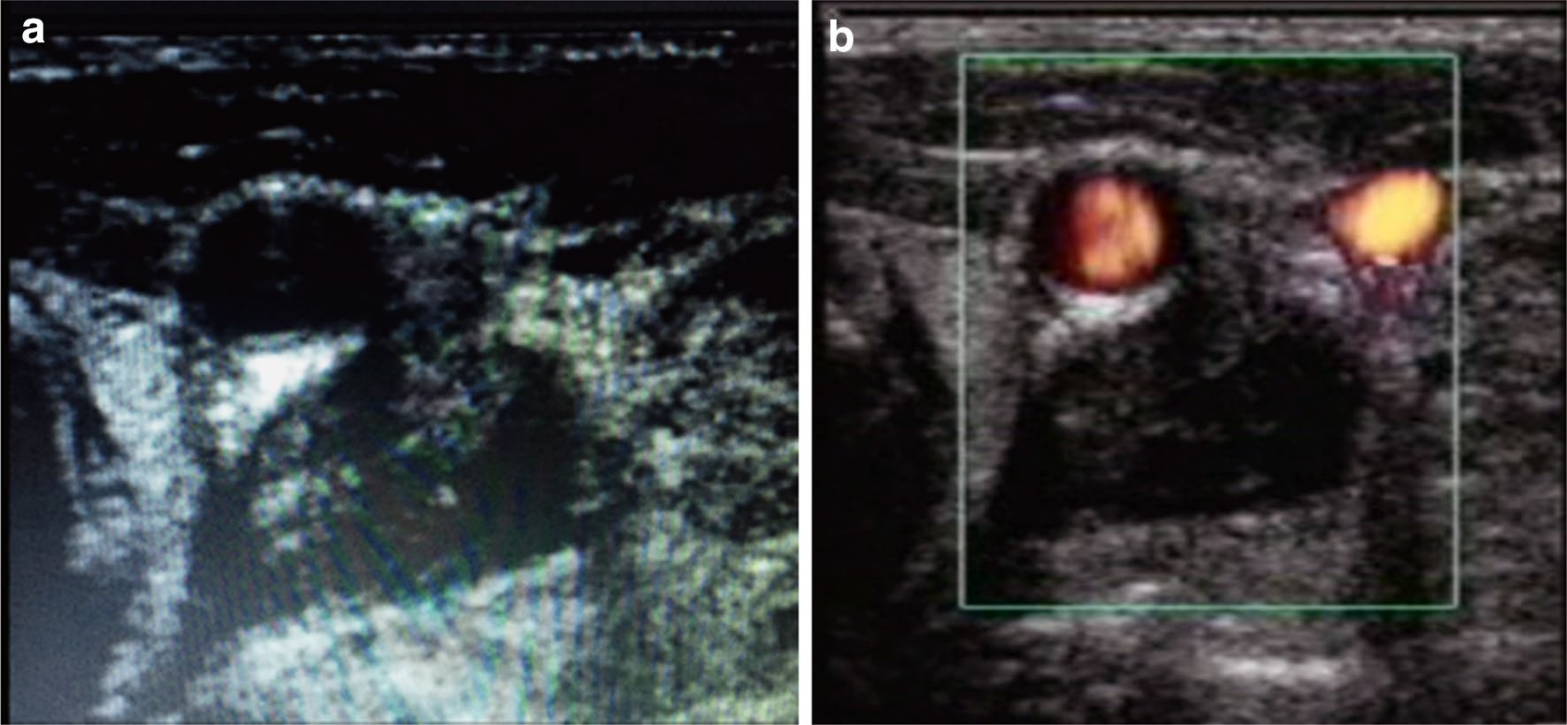
Perivascular hematoma. Knife penetrating wound in the* left side* of the neck**. a** Transverse view of the neck showing an hypoecoic hematoma between carotid and yugular vein. **b** Power Doppler showing indemnity of carotid and yugular with the hemathoma between the two vessels

Ultrasound can rapidly rule out vascular injuries after trauma at the bedside (Figs. 2, 3, 4).

When there is a trauma-related vascular lesion three basic patterns can be identified:

- Venous injuries
- Arterial injuries
- Mixed arterial and venous injuries

#### Venous injuries

**Fig. 5:**
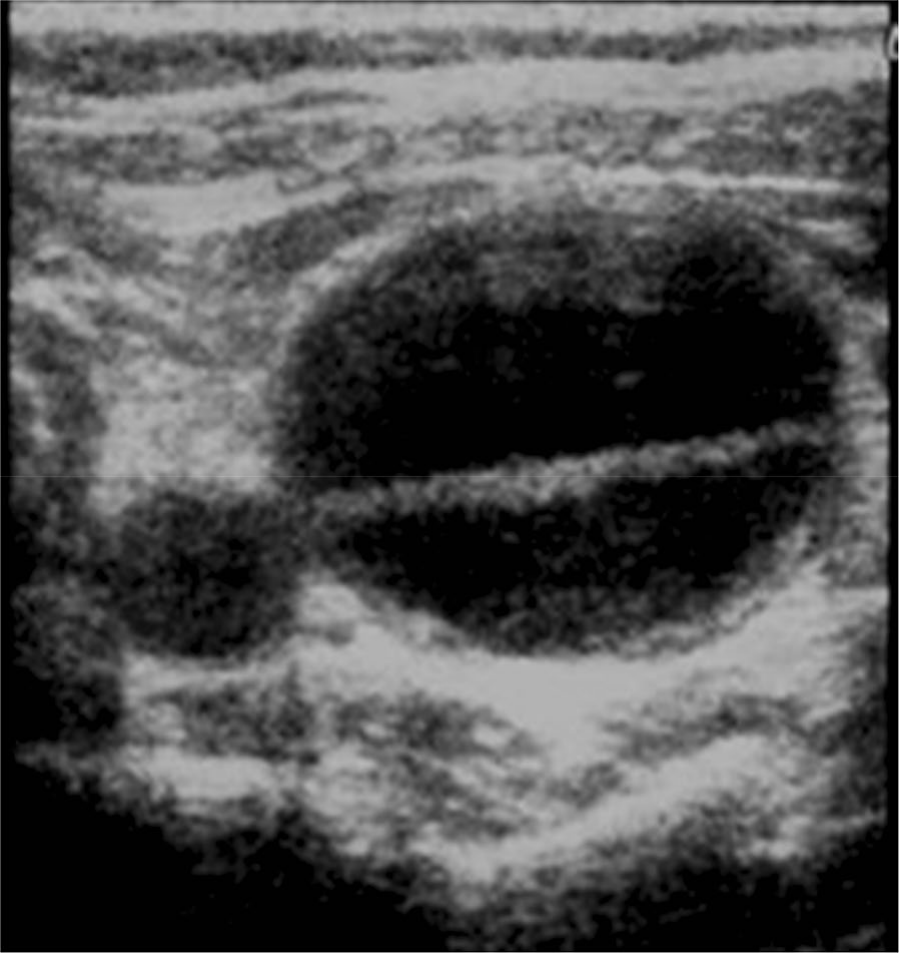
Blunt trauma to the neck. Transverse view of the *left side* of the neck showing the carotid artery and a dilated jugular vein with with detachment of the wall

Normal veins are anechoic and easily totally compressible by ultrasound transducers [21, 22, 25]. Traumatized veins may suffer partial or complete disruption, intimal injury, or post-traumatic thrombosis and this may alter the normal characteristics of these vessels. In complete disruption, it might be difficult to identify the site of injury due to shrinkage of injured veins or vein compression by the resultant hematoma. The presence of hematoma in the anatomical site where a major large caliber vein is supposed to be seen without a clear visualization of the vein is a strong indicator of complete vein rupture. In some blunt trauma patients, the ultrasonographic identification of a venous intimal flap injury may occur (Fig. 5).

Other common injury in trauma patients is vein thrombosis. When present, it usually appears after minutes or hours after the trauma. For the identification and diagnosis of venous thrombosis, we can use the compression technique and the Color Duplex Doppler evaluation [25–30].

**Fig. 6:**
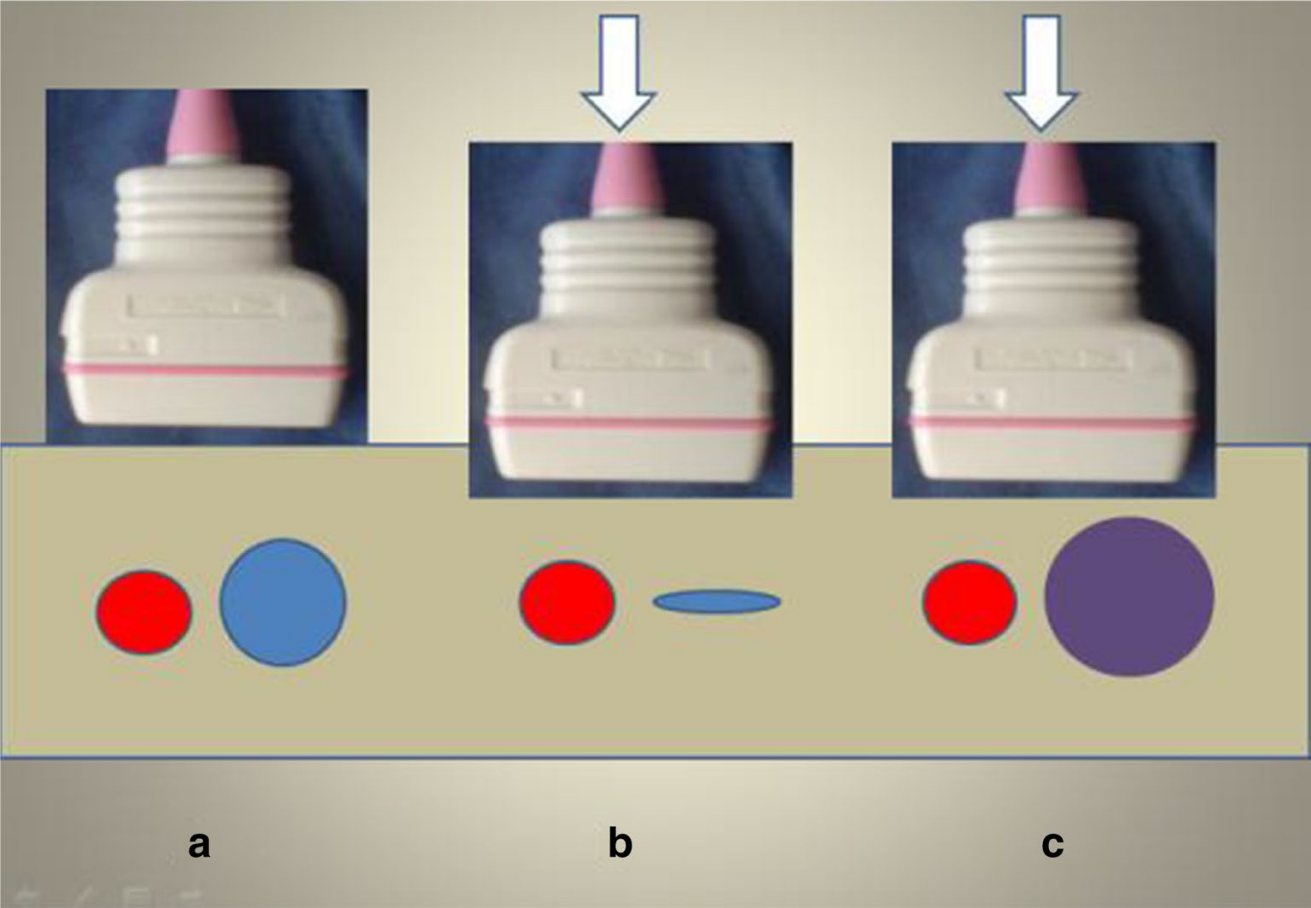
Schematic representation of the compression vein technique. **a** Without compression, **b** compressive normal vein, **c** non compressive venous thrombosis

**Fig. 7:**
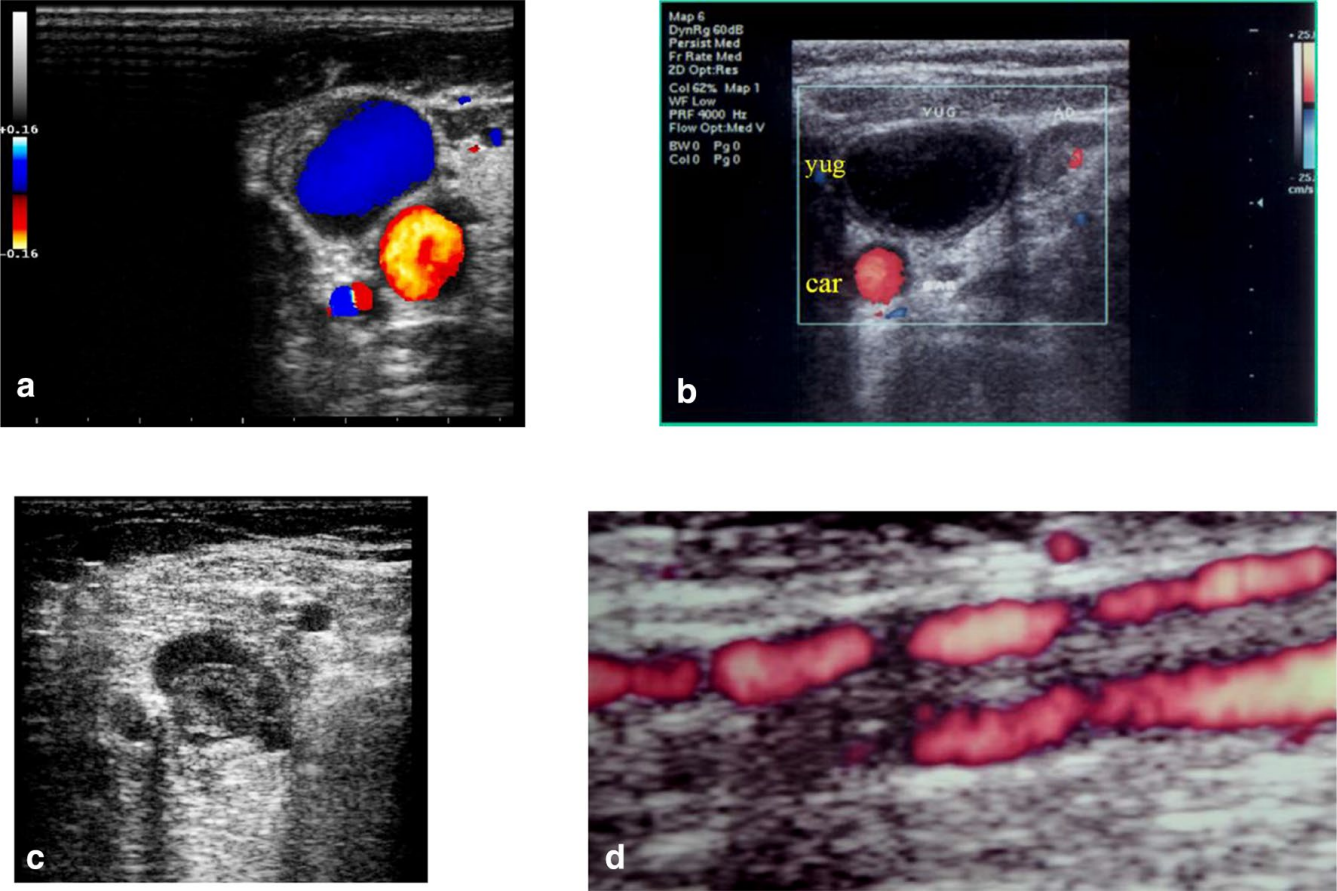
Venous thrombosis. **a** Partial thrombosis of right yugular vein: color Doppler (jugular vein in *blue*, carotid artery in *red*). **b** Complete acute hypoechoic thrombosis of left jugular vein (yug): transverse view. Carotid artery is seen in *red* (car). **c** Post trauma partial venous thrombosis. **d** Chronic thrombosis: partial recanalization: multiple color tunnels are seen inside the thrombus

In cases of acute thrombosis, the vein appears dilated, anechoic, and is not compressible (Fig. 6). In chronic thrombosis, the thrombus appears more echogenic and the vein is not dilated. Occasionally, in chronic thrombosis, the vein is partially collapsible, due to partial thrombosis or, in chronic thrombosis, because of the presence of channels within the thrombus, that leave the blood pass through (thrombus channeling or tunneling) (Fig. 7).

**Fig. 8:**
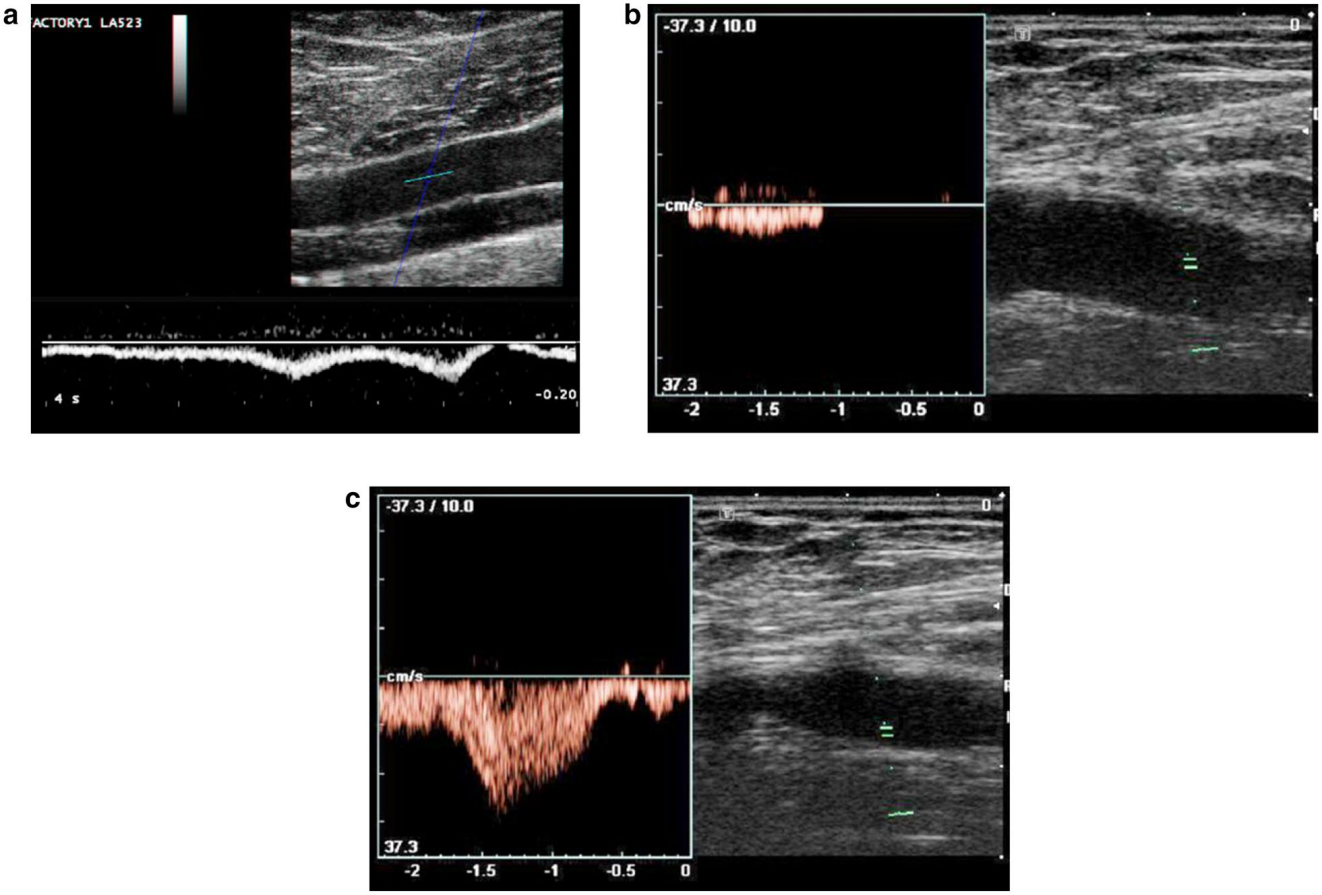
Normal spectral Doppler venous flow. **a** respiratory variability, **b** a proximal compression or a Valsalva maneuver cause stop of the flow, **c** the distal compression of the vein cause an increase of the velocity of the flow (augmentation)

Color Duplex Doppler evaluation will show the presence or absence of blood flow and the direction and velocity of the flow. In normal veins, the flow is continuous and spontaneous at rest and the velocity varies with respiration, increasing during inspiration, and decreasing during expiration or when a Valsalva maneuver is performed. The active compression of distal segments of the legs will cause an increase or “augmentation” of the velocity of the venous flow (Fig. 8). In case of partial thrombosis, the flow may be normal or pathologic, losing the variability during respiration. In total occlusive thrombosis, there will be complete absence of flow in color Doppler and in the spectral analysis.

#### Arterial injuries

**Fig. 9:**
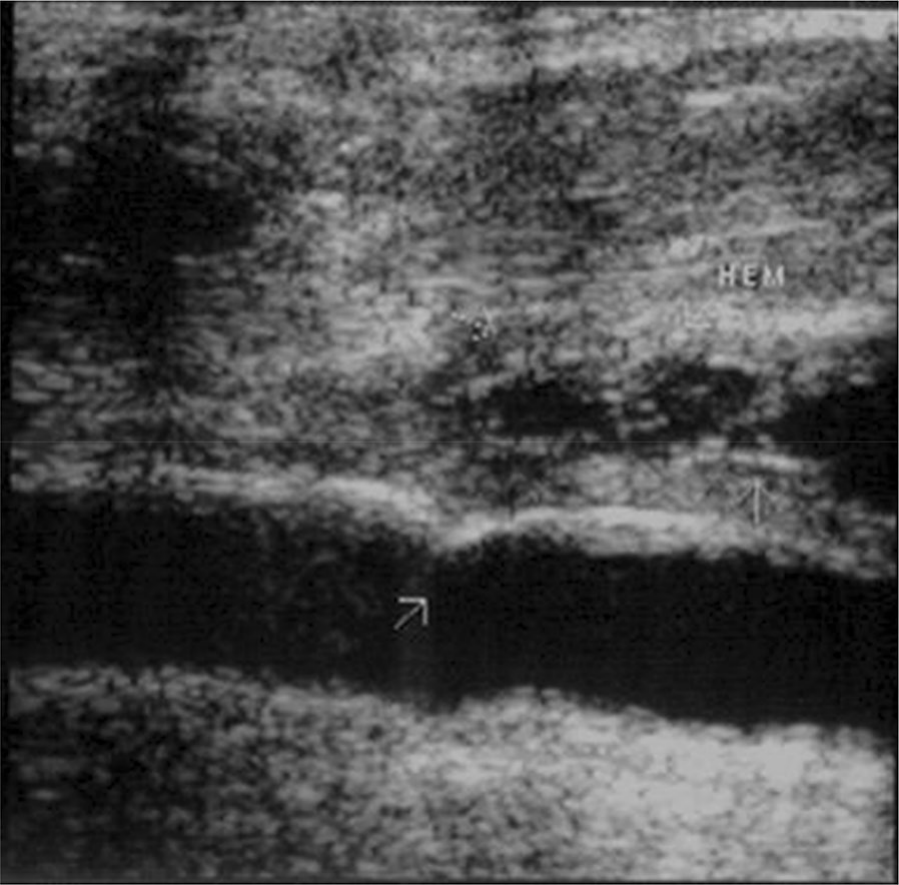
B mode of carotid artery after a penetrating wound caused by a knife: soft tissues edema and irregularity of the anterior wall of the carotid artery is clearly observed

In most cases, blunt and penetrating traumas cause only a slight wall irregularity (Fig. 9). When a serious injury occurs, intimal detachments, pseudoaneurysms, or arteriovenous fistula may occur.

**Fig. 10:**
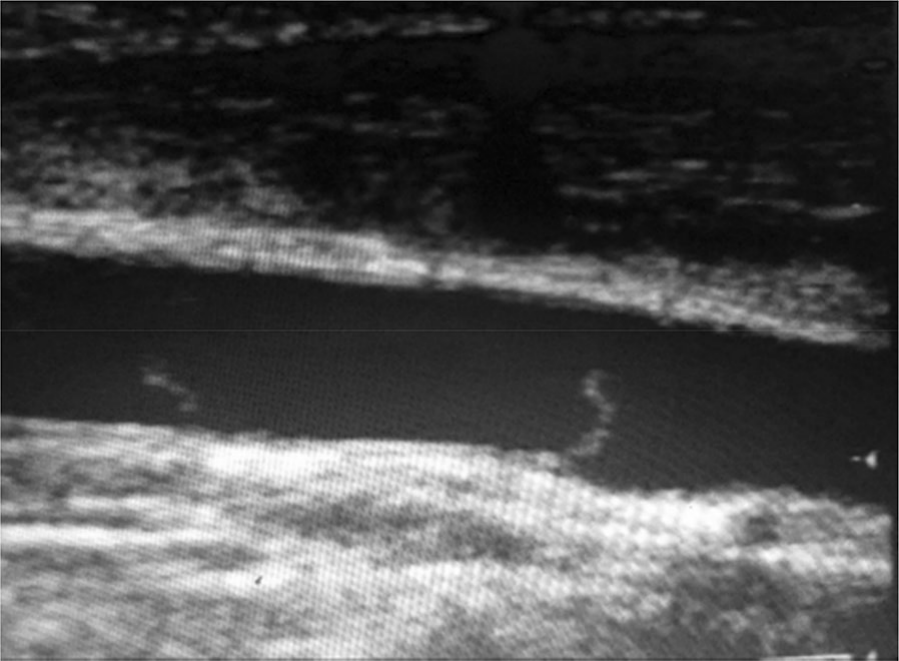
Post traumatic carotid artery intimal flap. B mode. Echogenic flap into the lumen

**Fig. 11:**
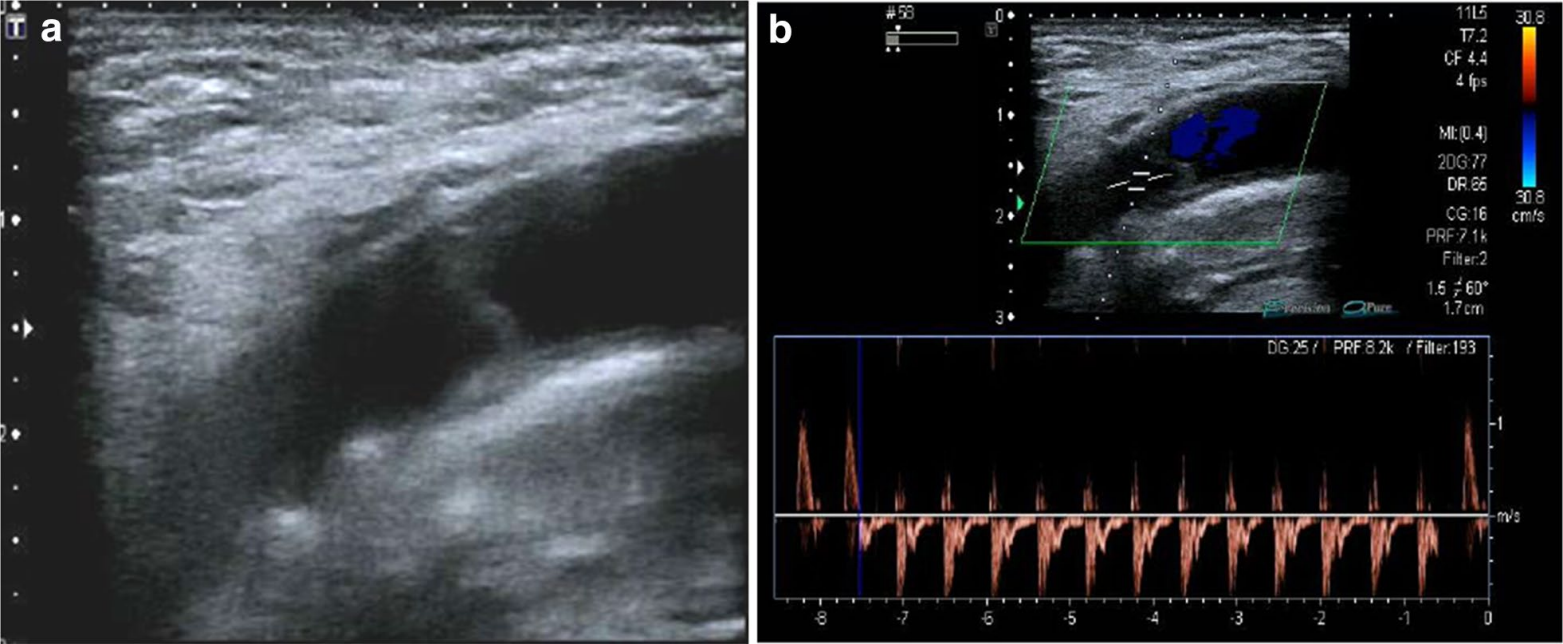
Post traumatic femoral artery intimal flap. **a** B mode. Small echogenic flap into the lumen. **b** Turbulent flow with change of direction of the flow was seen moving the sample volume near the moving flap

**Fig. 12:**
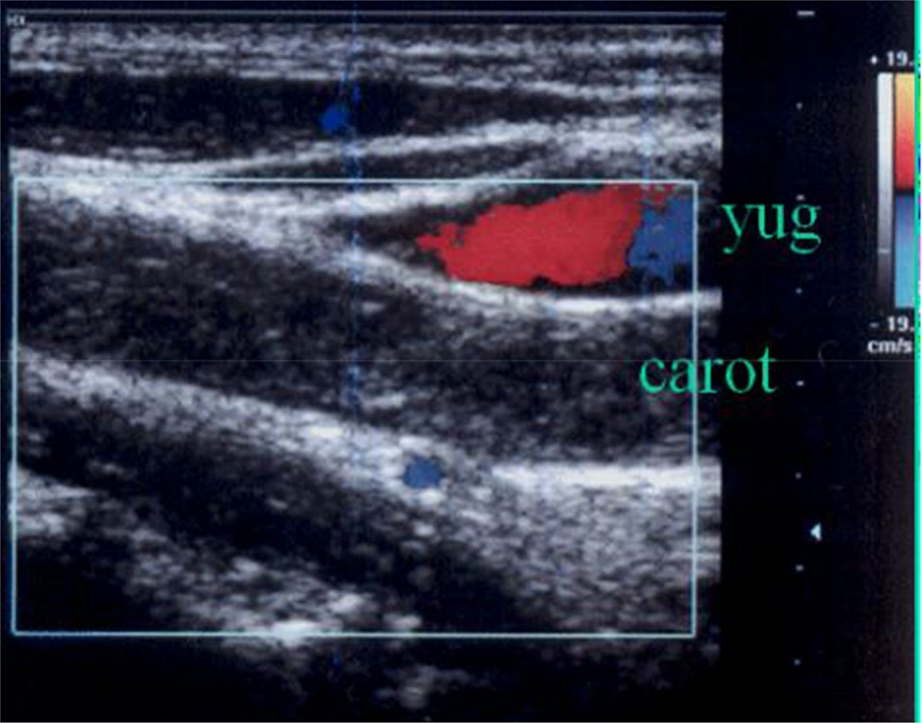
Complete post traumatic carotid artery thrombosis. Longitudinal view of the neck shows abscence of flow in the carotid artery (carot). The jugular vein is partially visualized (in *red* and *blue*)

Regarding ***intimal flaps***, they appear as thin, echogenic structures swimming within the vessel lumen (Fig. 10). In some cases, depending on the size of the intimal flap, PWD and CFD may show turbulence and irregular flow (Fig. 11). The usual approach for most cases is to *wait and see*, supplemented or not with antiplatelet medication, and a strict follow-up [31, 32]. Endovascular management of intimal defects has also been proposed [32] Occasionally, these injuries can progress to arterial thrombosis. The presence of arterial thrombosis is observed in B-mode as echogenic material occupying arterial lumen. Doppler examination may show no flow both in PWD and CFD mode (Fig. 12).

**Fig. 13:**
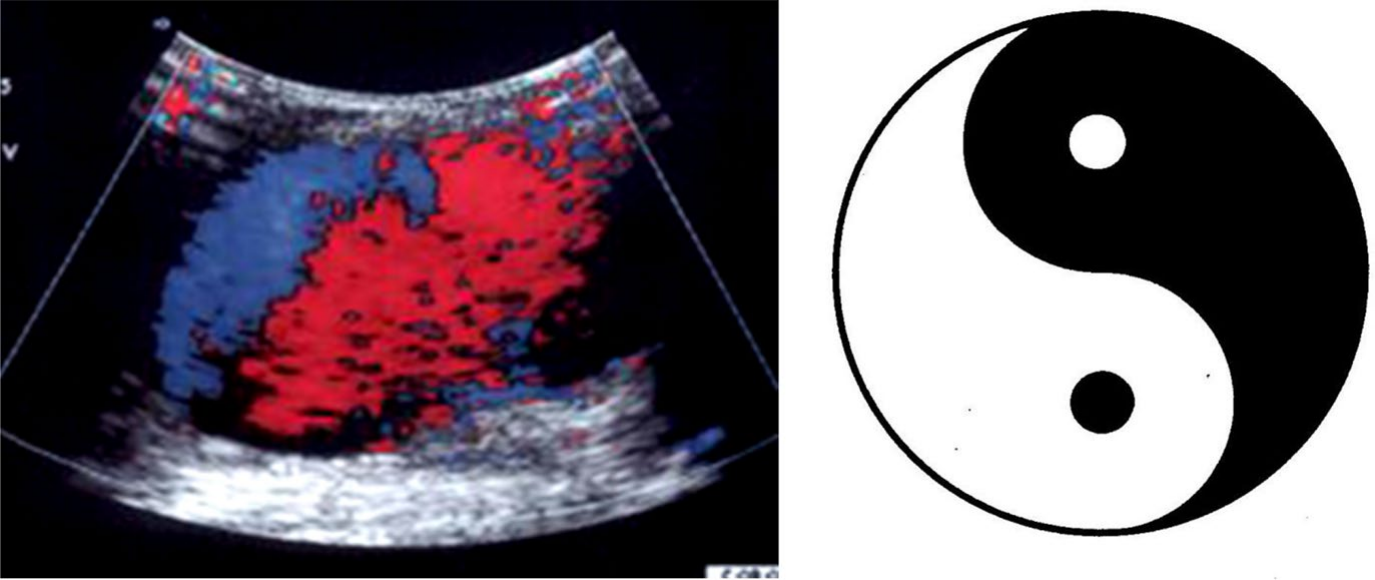
Pseudoaneurism: color Doppler aspect: colorized yin yang sign

**Fig. 14:**
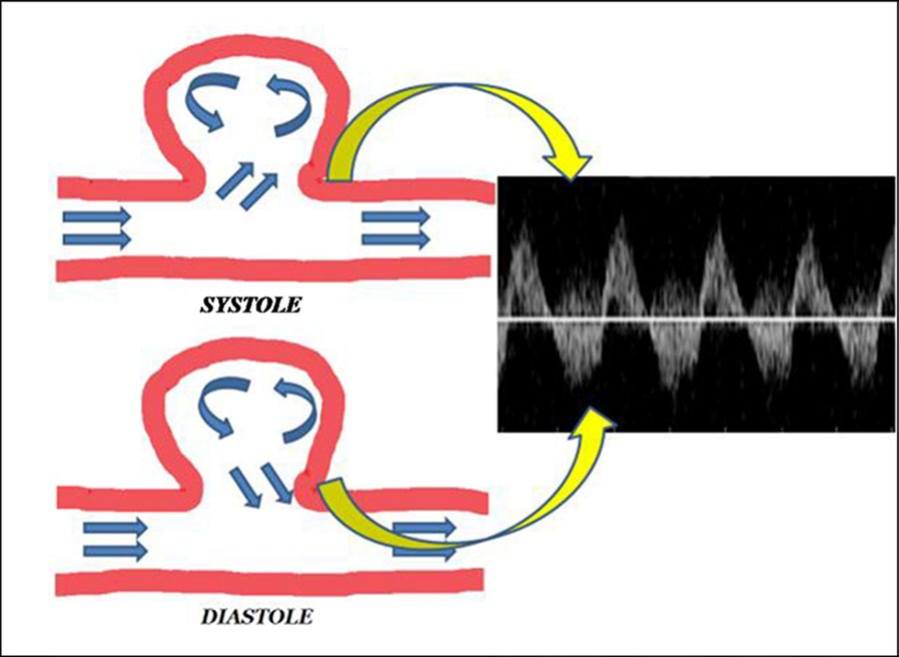
Schematic representation of the movement of the flow into a pseudoaneurism causing a characteristic biphasic flow in the neck of the PA

**Fig. 15:**
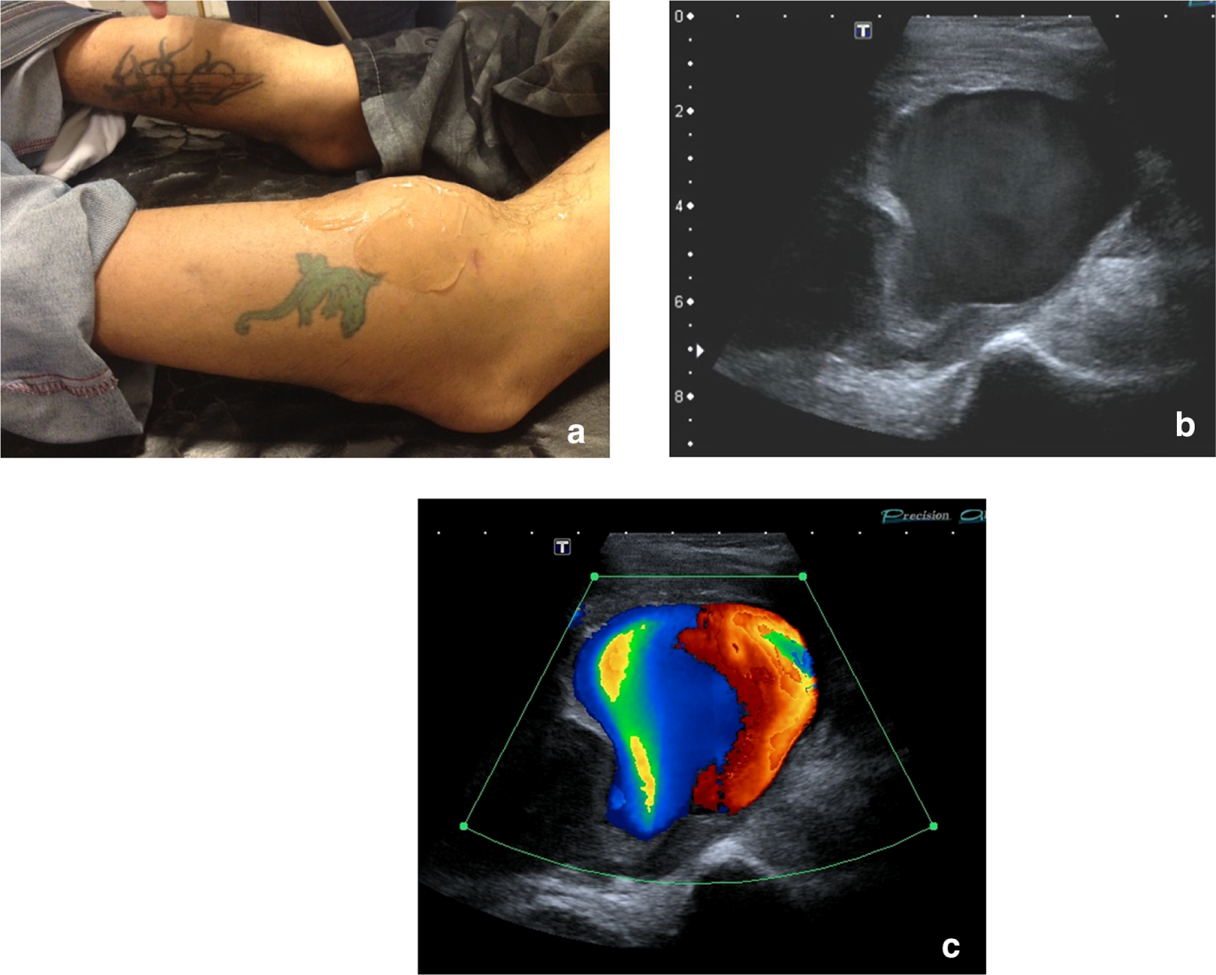
Popliteal pseudoaneurism (gun shot wound one week before). **a** Left popliteal mass. **b** B mode: hipoechoic liquid image with swirling movements in real time. **c** Color Doppler: Yin Yang colorized sign

**Fig. 16:**
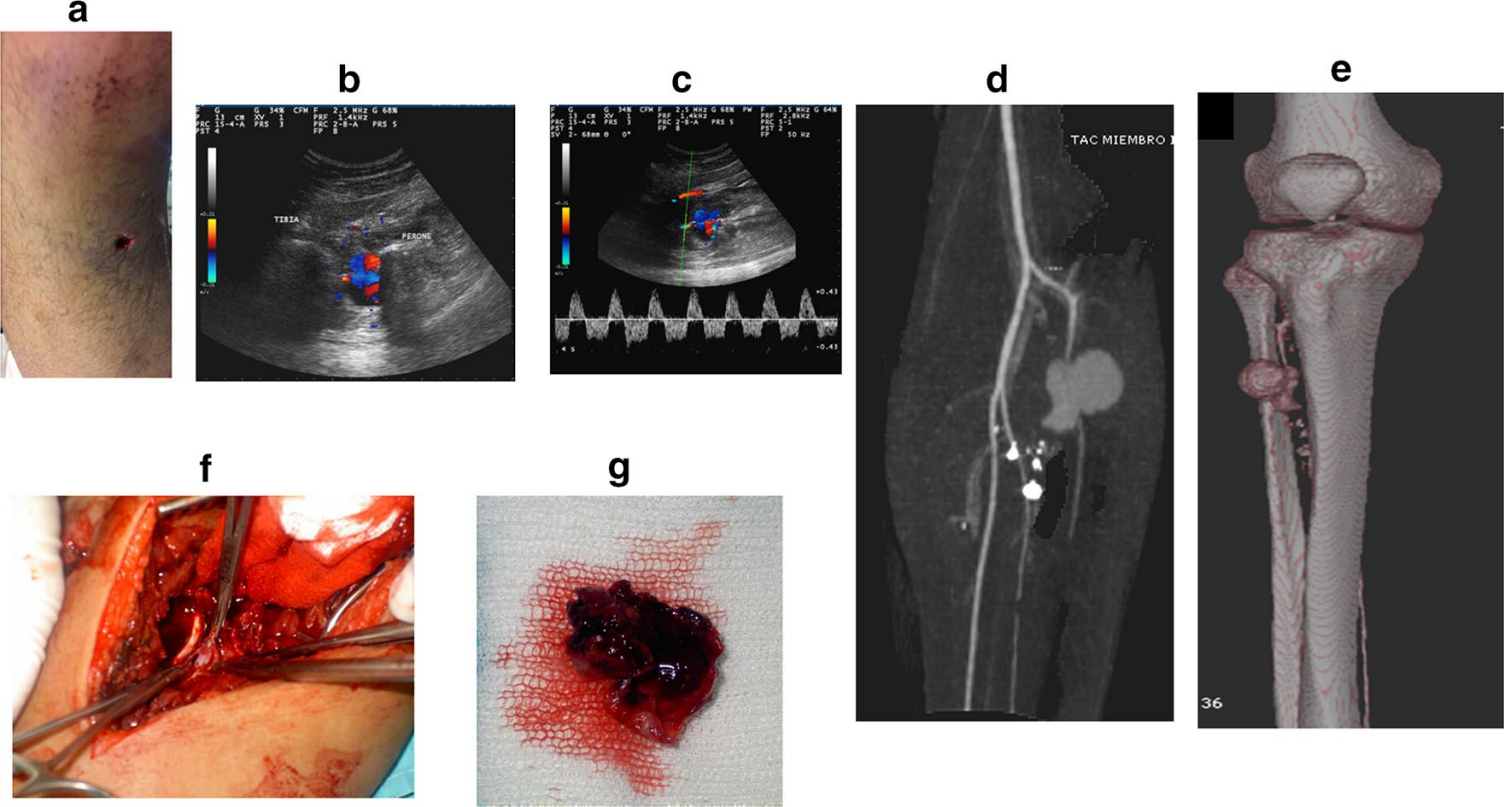
Pseudoaneurism: Gun shot gun wound in the *right leg* with an arterial tibial pseudoaneurism. **a** Penetrating wound, **b** pseudo aneurism: color Doppler, **c** pulsed Doppler with the sample volume at the neck of the pseudoaneurism: bidirectional flow (to and fro), **d** CT angiography showing the pseudoaneurism and the metallic foreign bodies, **e** CT: 3D reconstruction, **f** surgical repairment, **g** intraoperative photography of the resected pseudoaneurysm

A ***pseudoaneurysm*** is produced by the partial rupture of an artery wall, which causes accumulation of periarterial blood, surrounded and contained by the adventitia, hematomas, or soft tissue. It is always in communication with the arterial lumen by a small portion called pseudoaneurysm neck. The most common cause is an interventional medical procedure [15, 33]. Penetrating trauma, by gunshot or stab wound, is other very common cause [34, 35]. Using the B-mode, the presence of an anechoic or hypoechoic image, similar to a hematoma, but containing moving echoes (pulsatile hematoma), may be observed. Occasionally, it can swell during systole. The lumen can be partially filled by echogenic structures due to the presence of thrombus. When using CFD evaluation, the diagnosis is made by showing the typical swirling motion that causes a colorized yin–yang sign (Fig. 13). This sign is cause by the circular motion of the blood inside the pseudoaneurysm cavity. During systole, the flow goes towards the pseudoaneurysm cavity (red) and during diastole the blood moves back to the arterial lumen (blue) [33, 35]. Inside the pseudoaneurysm, turbulent flow is recorded with the spectral analysis. If we place the sample volume on the neck of the pseudoaneurysm, we might be able to see a characteristic flow pattern known as “to-and-fro.” This is a bidirectional flow caused by the continuous entry and exit of blood from the artery to the pseudoaneurysm (Figs. 14, 15, 16). Ultrasound is also used to treat of iatrogenic femoral pseudoaneurysm. Ultrasound-assisted arterial compression, thrombin injection, and further monitoring have demonstrated to have high efficacy and low complications rates [36, 37].

#### Mixed arterial and venous injuries

**Fig. 17:**
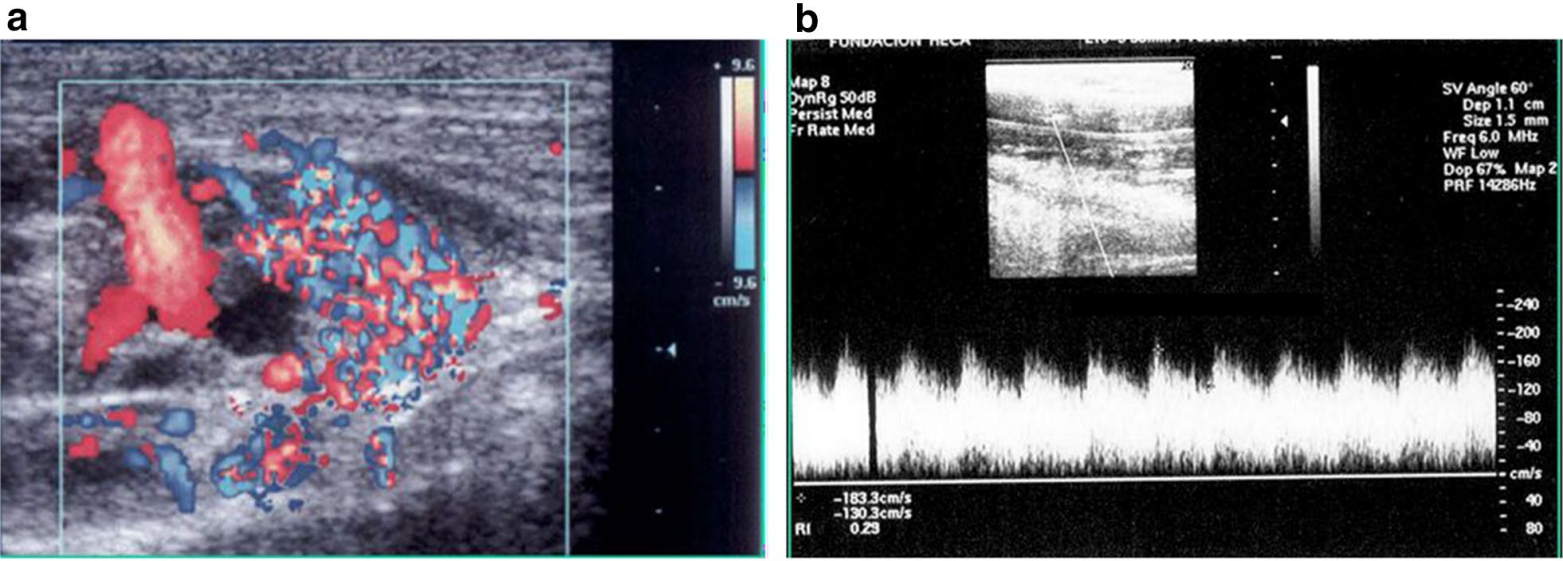
Arterio venous fistula. **a** Color Doppler aspect: mosaic of colours representing multiple different velocities. **b** Spectral analysis: high systolic and diastolic velocities

**Fig. 18:**
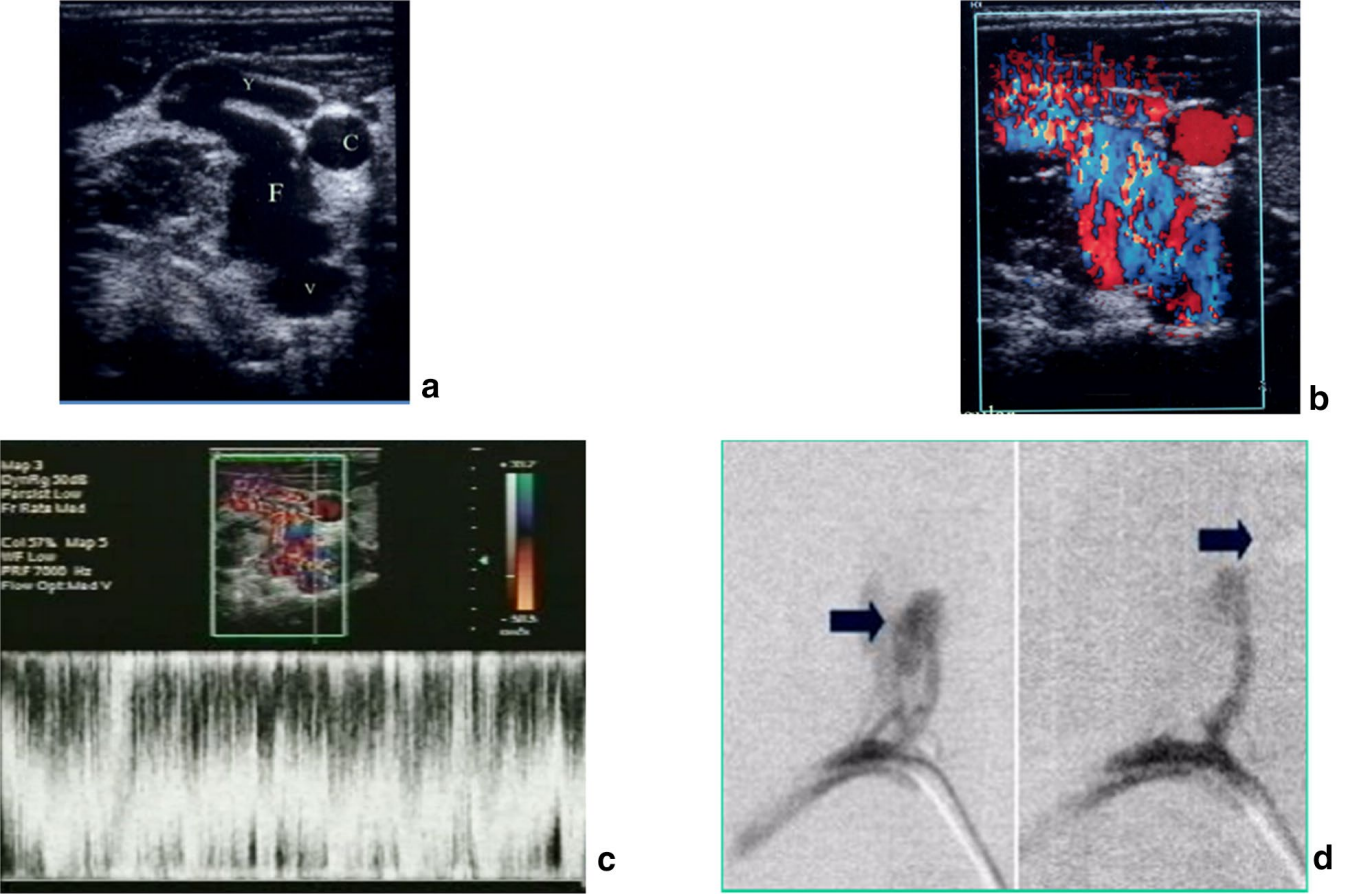
Arterio venous fistula: gun shot wound in the* right side* of the neck. Arteriovenous traumatic fistula between right yugular vein and right vertebral artery. **a** B mode: anechoic image connecting right yugular vein with the right vertebral artery. *C* carotid artery, *V* vertebral artery, *Y* yugular vein, *F* fistula. **b** Color Doppler: mosaic of colours representing different velocities. Color in soft tissues surrounding the FAV representing the thrill. **c** Pulse Doppler: spectral analysis with hight velocities spectrum and turbulent flow. **d** Angiographic findings

An ***Arteriovenous fistula*** (AVF) is a permanent communication between an artery and a vein. Depending of the size, it may cause hemodynamic alterations with cardiac effects [38, 39]. It can be congenital or acquired (i.e., traumatic, iatrogenic) [40, 41]. The vein caliber and local venous pressure usually increases due to different pressure between arteries and the veins. These modifications are followed by a phenomenon called arterialization of the flow and the vessel. It is unusual to diagnose AVF soon after trauma. A murmur and thrill on the site of the fistula may be present. With B-mode, the presence of an anechoic, anfractuous structure communicating the vein and artery may be seen. CFD examination may reveal the presence of a color mosaic (aliasing), due to the presence of multiple velocities inside the fistula, causing high flow turbulence. The pulsed Doppler spectral analysis may show high systolic and diastolic velocities with loss of systolic windows due to the wide velocity range (Figs. 17, 18).

### Distal flow analysis

**Fig. 19:**
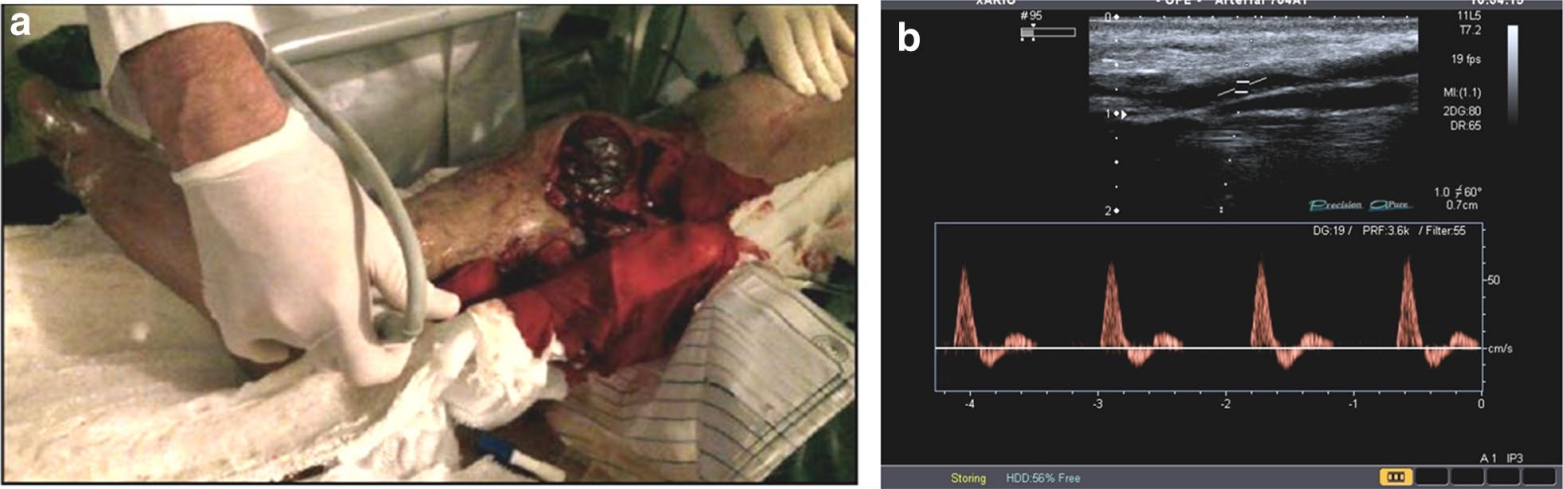
Open fracture of the tibia. **a** Displaced long bone fracture with segment exposure. Exploration of distal pulses. **b** Normal triphasic flow

**Fig. 20:**
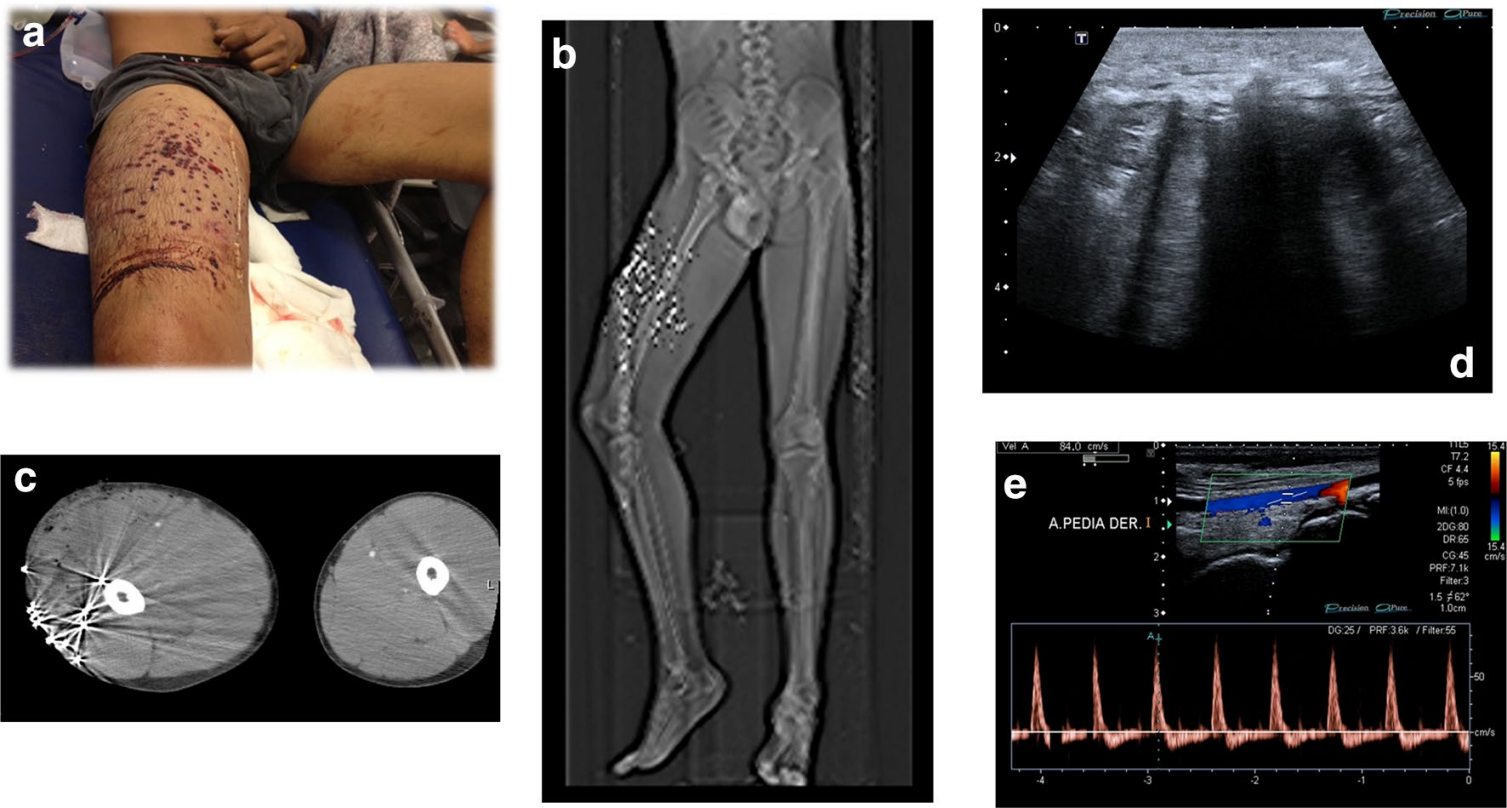
External compression. **a** Multiple penetrating gun shot wounds in the right tight. **b** CT scan. **c** CT multiple bullets. Increase diameter of the right tight. **d** Ultrasound showed the metallic foreign bodies with acoustic shadow and edema of the soft tissues. **e** Color Doppler showed no vascular lesion in the right tight Pulse wave Doppler showed lost of the triphasic normal flow in distal arteries caused by external compression of the arteries cause by edema and tumefaction

The presence of triphasic flow in the distal arteries of the lower extremities may be interpreted as an indication of absence of lesion in the proximal arteries [21] (Fig. 19). Arterial injuries of the lower limbs, (being acute or chronic) can cause distal alteration of the flow (Fig. 20). The absence of flow or the presence of a biphasic or monophasic flow may be considered pathologic and further investigation may be necessary [42]. If an arterial injury is suspected, the detection of triphasic flow in distal arteries of the lower limbs may help to rapidly rule out a significant vascular injury with 100% sensitivity (Montórfano et al. The FAST D protocol: A simple method to rule out traumatic vascular injuries of the lower extremities. Submitted to Critical Ultrasound Journal).

#### Eco Doppler ultrasound monitoring

**Fig. 21:**
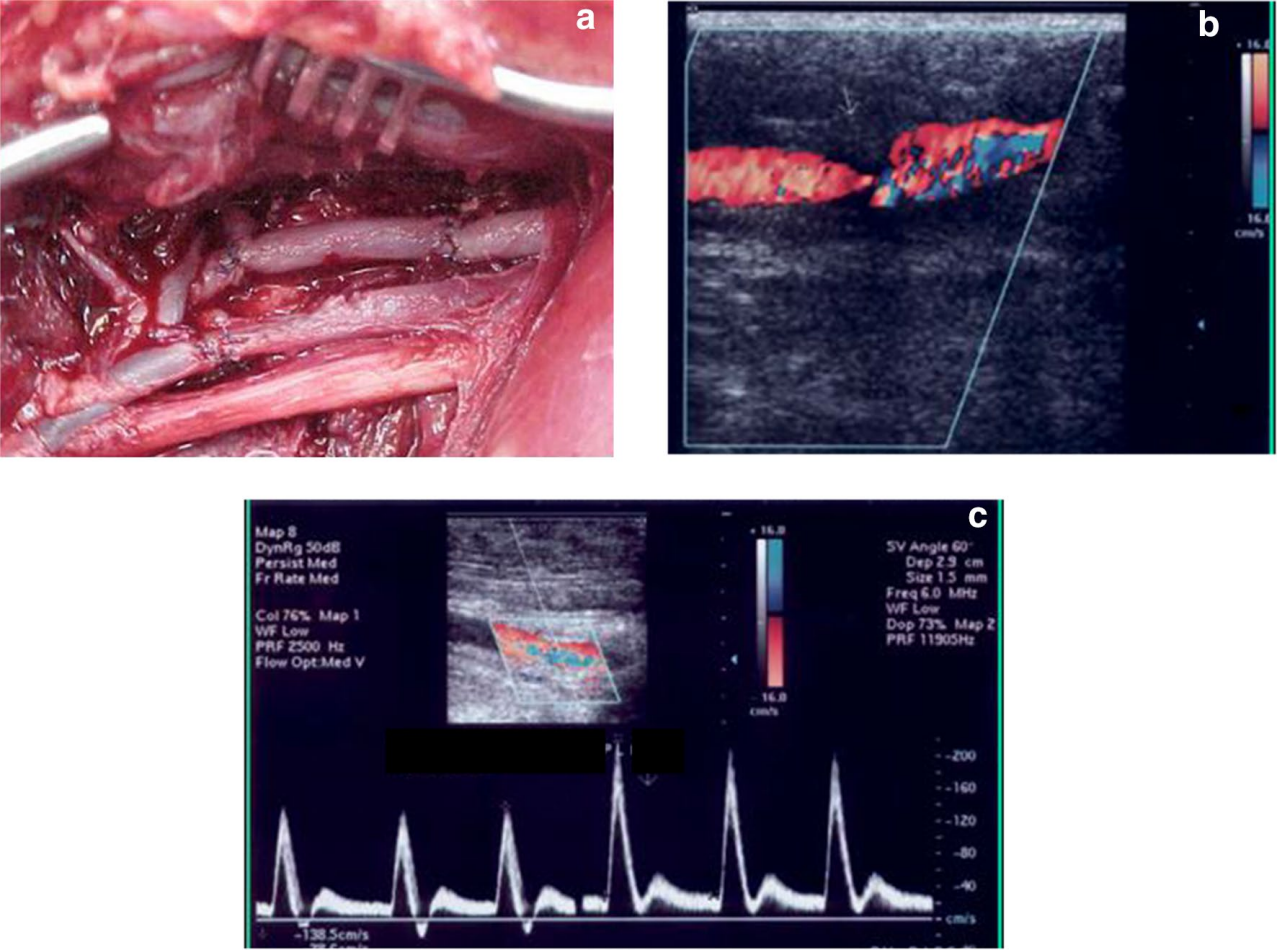
Gun shot femoral artery injury. Post operative follow up. **a** Surgical repairment with vein interposition. **b** Color flow: stenosis with turbulent flow in the anastomosis. **c** Increase of the velocity at the level of the stenosis in the pulsed Doppler spectrum

B-mode ultrasound and Color flow Duplex Doppler ultrasound can also be used for monitoring and follow-up after endovascular or surgical treatment of vascular injuries [41, 43–46]. B-mode evaluation is useful for monitoring the soft tissue surrounding the lesion, and for ruling in and ruling out hematomas and fluid collections. The Color flow Duplex Doppler technique is used for the evaluation of blood flow patterns in the anastomotic area, proximal and distal flow characteristics, the presence of pathological flow variation, and eventual occlusions [21] (Fig. 21).

## Conclusions

When there are no hard signs of vascular injury and the patient is stable, imaging methods can aid in the localization and diagnosis of trauma-related vascular lesions. While angiography still remains the gold standard for the assessment of traumatic vascular injuries of the limbs and neck, other less invasive imaging modalities have gained popularity in the recent years and are routinely used. Point-of-care ultrasound and Color flow duplex Doppler ultrasound are widely available, noninvasive, sensible and specific techniques that can be used bedside, as a first approach for a prompt diagnosis and follow-up of trauma-related vascular injuries, and for an integrated management of trauma patients.
